# Sirt1 Mediates Vitamin D Deficiency-Driven Gluconeogenesis in the Liver via mTorc2/Akt Signaling

**DOI:** 10.1155/2022/1755563

**Published:** 2022-01-29

**Authors:** Qi Yuan, Ridong Zhang, Mengyue Sun, Xiao Guo, Jinglei Yang, Wen Bian, Chunfeng Xie, Dengshun Miao, Li Mao

**Affiliations:** ^1^Department of Endocrinology, The First Huaian Hospital Affiliated to Nanjing Medical University, Huai'an, 223300 Jiangsu, China; ^2^Nanjing Medical University, School of Public Health, Nanjing, 210000 Jiangsu, China; ^3^Nanjing Medical University, School of Basic Medicine, Nanjing, 210000 Jiangsu, China

## Abstract

As an active form of vitamin D (VD), 1,25-dihydroxyvitamin D (1,25(OH)_2_D_3_) is involved in the development of many metabolic diseases, such as diabetes, autoimmune diseases, and tumours. While prospective epidemiological studies have consistently implicated VD deficiency in the regulation of glucose metabolism and insulin sensitivity, the specific mechanism remains unclear. Here, we generated 1*α*(OH)ase-null mice (targeted ablation of the 25-hydroxyvitamin D 1*α* hydroxylase enzyme) and found that these mice developed hepatic glucose overproduction, glucose intolerance, and hepatic insulin resistance accompanied by reduced Sirtuin 1 (Sirt1) expression. The chromatin immunoprecipitation (ChIP) and a luciferase reporter assay revealed that 1,25(OH)_2_D_3_-activated VD receptor (VDR) directly interacted with one VD response element (VDRE) in the Sirt1 promoter to upregulate Sirt1 transcription, triggering a cascade of serine/threonine kinase (AKT) phosphorylation at S473 and FOXO1 phosphorylation at S256. This phosphorylation cascade reduced the expression of gluconeogenic genes, eventually attenuating glucose overproduction in the liver. In addition, a signaling pathway was found to modulate gluconeogenesis involving VDR, Sirt1, Rictor (a component of mTOR complex 2 [mTorc2]), AKT, and FOXO1, and Sirt1 and FOXO1 were identified as key modulators of dysregulated gluconeogenesis due to VD deficiency.

## 1. Introduction

Type 2 diabetes is caused by a combination of genetic and environmental factors. Long-term observational studies have consistently linked vitamin D (VD) deficiency or VD receptor (VDR) gene polymorphism to impaired glucose metabolism [1–4], although Pittas et al. reported in 2019 that extra VD supplementation did not contribute to the prevention of diabetes regardless of the baseline serum 25-hydroxyvitamin D level [5]. In addition to the small amount of VD absorbed by the intestine, the main source of VD is synthesis in the skin. VD forms 25-hydroxyvitamin D under the action of 25-hydroxylase in the liver, which is then converted into the active form of VD, 1,25-dihydroxyvitamin D_3_ [1,25(OH)_2_D_3_], by the action of the 1*α*-hydroxylase enzyme in the kidney. 1,25(OH)_2_D_3_, a nuclear hormone, modulates gene transcription and exerts its effects through directly binding its receptor, VDR, which forms a complex with the retinoic acid receptor (RXR) to act on various VD response elements (VDREs) located in the DNA promoter [6]. Type 2 diabetes is primarily characterized by abnormal hepatic gluconeogenesis and insulin resistance; nevertheless, the mechanism by which VD regulates gluconeogenesis under physiological conditions should be further investigated.

FOXO1, a transcription factor primarily regulated through its phosphorylation on multiple residues, plays important roles in the modulation of gluconeogenesis and glycogenolysis through insulin/Akt signaling [7, 8]. Feeding can invoke insulin signaling in the liver by activating the phosphorylation of serine/threonine kinase (AKT) and downstream FOXO1, which results in the exclusion of FOXO1 from the nucleus, inducing the suppression of fasting-induced transcription of glucose-6-phosphatase (G6Pase) and phosphoenolpyruvate carboxykinase (PCK1), thus reducing gluconeogenesis [9]. FOXO1 has been reported to mediate VD deficiency-induced insulin resistance in the skeletal muscle [10]; however, the mechanistic links between VD signaling, gluconeogenesis, and insulin resistance in the liver remain unclear. The enzyme Sirtuin 1 (SIRT1) which is a member of the sirtuin protein family is responsible for deacetylating the proteins critical for cellular regulation [11, 12], and its gain of function increases energy efficiency and prevents diabetes in mice [13]. It has been reported that liver-specific deletion of Sirt1 upregulates the expression of G6Pase and PCK1 by impairing the mTorc2/AKT/FOXO1 pathway and thus increases hepatic glucose production and chronic hyperglycaemia [14]. Our preliminary experiments revealed that 1,25(OH)_2_D_3_-deficient mice had downregulated expression of Sirt1. However, whether VD deficiency affects gluconeogenesis and insulin resistance in the liver through regulating Sirt1 expression and AKT/FOXO1 signaling remains unclear.

Here, we investigated the impact of 1,25(OH)_2_D_3_ deficiency on the gluconeogenesis with deletion of 1*α*(OH)ase *in vivo* and in VDR-knockdown HepG2 cells *in vitro*. Our results indicated that 1,25(OH)_2_D_3_ positively regulated the expression of Sirt1, an important deacetylase engaged in modulating glucose and lipid metabolism, by binding to its receptor, VDR. Reduced Sirt1 levels in 1*α*(OH)ase^−/−^ mice downregulated the expression of Rictor, a key component of the mTorc2 complex, impaired the phosphorylation of AKT at S473 and FOXO1 at S256, and increased the expression of G6pase and PCK1, which resulted in hepatic glucose overproduction, hepatic insulin resistance, and, eventually, chronic hyperglycaemia.

## 2. Materials and Methods

### 2.1. Animals and Treatments

The strain of all mice used in our study was C57BL/6J. The 1*α*(OH)ase^−/−^ mice were generated and characterized as previously described by Panda et al. [15]. Age- and sex-matched 1*α*(OH)ase^−/−^, 1*α*(OH)ase^−/−^Sirt1^Tg^, and wild-type (WT) littermates were randomly divided into four groups (>10 mice per group). To avoid the effects of hormones, all the mice used in this study were male. After weaning, male WT, 1*α*(OH)ase^−/−^, and 1*α*(OH)ase^−/−^Sirt1^Tg^ mice were divided into one of the following different groups: (1) WT mice were fed with a rescue diet (RD) (containing 2.0% calcium, 1.25% phosphorus, and 20% lactose), (2) 1*α*(OH)ase^−/−^ mice were fed with a RD, (3) 1*α*(OH)ase^−/−^ mice were fed with a normal diet (ND) (containing 1.0% calcium and 0.67% phosphorus) and subcutaneously injected with 1,25(OH)_2_D_3_ (Sigma, #705942, the normal diet plus three weekly subcutaneous injections of 1,25(OH)_2_D_3_ at a dose of 1 *μ*g/kg per mouse), and (4) 1*α*(OH)ase^−/−^Sirt1^Tg^ mice were fed with a RD. All animal experiments were approved by the Animal Care and Use Committee of Nanjing Medical University.

### 2.2. Measurements of Serum Calcium and Phosphorus Levels

Serum calcium and phosphorus levels were analysed with an autoanalyser (Hitachi 7180, Japan) using corresponding kits from MedicalSystem Biotechnology Co., Ltd.

### 2.3. Glucose Measurement

The mice were maintained under feeding or fasting conditions. The blood glucose level in the experiments was measured with a glucose meter (Bayer) and corresponding glucose test strips (Bayer).

### 2.4. Pyruvate Tolerance Test (PTT)/Glucose Tolerance Test (GTT)

After fasting for 16 hours, the body weight and blood glucose level were measured, and 20% pyruvate (PTT, Sigma, #792500)/20% glucose (GTT) was injected into each mouse i.p. at 100 *μ*L/10 g body weight. The blood glucose level was monitored with a glucose meter at 15- or 30-minute intervals over 2 hours. Meanwhile, the blood was collected at 0, 15, and 120 minutes after glucose injection during the GTT, and the insulin level was measured.

### 2.5. Insulin Tolerance Test (ITT)

After measuring the body weight and glucose level, insulin was injected into each mouse fasting for 4 hours i.p. at 0.8 *μ*L/kg body weight. The blood glucose level was measured as in GTT/PTT.

### 2.6. Enzyme-Linked Immunosorbent Assay

Mouse serum 1,25(OH)_2_D_3_ and insulin levels were assayed by enzyme-linked immunosorbent assay with a mouse 1,25(OH)_2_D_3_ ELISA kit (Cusabio, #CSE-E1369m) and rat/mouse insulin ELISA kit (Millipore, #EZRMI-13K) following the manufacturer's instructions.

### 2.7. Cell Culture

HepG2 human hepatoma cells obtained from Cobioer Biosciences (Nanjing, China) were cultured in high-glucose Dulbecco's modified Eagle medium (DMEM) (Gibco, Carlsbad, CA, USA) containing 10% fetal bovine serum (Gibco), 100 IU/mL penicillin, and 100 *μ*g/mL streptomycin at 37°C in a humidified atmosphere containing 5% CO_2_.

### 2.8. RNAi Transfection

Control siRNA (si-NC) and si-VDR (si-VDR1, si-VDR2) for VDR knockdown were purchased from the GenePharma Company (Shanghai, China). The siRNA target sequence was 5′-GUGCCAUUGAGGUCAUCAUTT-3′. Cells were plated in 6-well plates and grown to a cell density of 70%. Then, transient transfection was performed using Lipofectamine RNAiMAX (Invitrogen, #13778-075) according to the manufacturer's instructions.

### 2.9. VD and SRT1720 HCL Treatment

HepG2 cells were grown with high-glucose DMEM containing 10% fetal bovine serum (FBS) in the absence or presence of 10^−7^ M 1,25(OH)_2_D_3_ (Sigma-Aldrich, #705942) for 48 hours. In addition, after transfection for 48 hours, HepG2 cells were maintained with high-glucose DMEM containing 10% FBS in the presence of 100 nM SRT1720 HCL (a selective SIRT1 activator, Selleck, #S1129) for another 6 hours for a subsequent glucose production assay with the supernatant.

### 2.10. Glucose Production Assay

HepG2 cells were cultured for 48 hours, washed in PBS to remove glucose, and then incubated in glucose production buffer [2 mM pyruvate and 20 mM lactate (Sigma, #1614308) in PBS]. Then, 100 nM insulin was added to the culture and incubated for 30 minutes before the samples were collected. Afterwards, glucose concentrations were measured 6 hours later with a glucose assay kit (Invitrogen, #A22189). The glucose concentration was calculated based on the difference between cells cultured in lactate/pyruvate-free medium and those cultured in pyruvate/lactate-supplemented medium.

### 2.11. Western Blotting

Western blotting was carried out with Tiangen using antibodies against VDR (Proteintech, #67192-1-Ig), Sirt1 (Cell Signaling Technology, #9475), Rictor (Cell Signaling Technology,#9476), FOXO1 (Cell Signaling Technology, #2880), FOXO1 phosphorylated at S256 (Cell Signaling Technology, #84192), AKT (Cell Signaling Technology), AKT phosphorylated at S473 (Cell Signaling Technology, #4060), G6pase (Bioss, #bs-4044R), PCK1 (Abcam, ab70358), and GAPDH (Proteintech, #60004-1-Ig). Immunoprecipitation was carried out according to standard procedures.

### 2.12. Immunohistochemistry

Immunohistochemical staining for FOXO1 phosphorylated at S256, AKT phosphorylated at S473, and Sirt1 was carried out using the avidin-biotin-peroxidase complex technique with antibodies against FOXO1 phosphorylated at S256 (Cell Signaling Technology, #84192), AKT phosphorylated at S473 (Cell Signaling Technology, #4060), and Sirt1 (Abcam, #ab189494).

### 2.13. Quantitative Real-Time PCR (qRT-PCR)

Total RNA was isolated from liver tissues or HepG2 cells with a TRIzol reagent (Invitrogen, #12183555). cDNA was synthesized using the PrimeScript RT reagent (Takara, #639506) reverse transcription kit. Quantitative RT-PCR was performed using the SYBR Green PCR Master Mix (Applied Biosystems, #4309155) and analysed using the ABI 7500 real-time PCR system. The primers used are shown in supplemental Table 1.

### 2.14. Luciferase Assay

The plasmids of Luc-SIRT1 and VDR overexpression were transfected into 293T cells. Two VDR binding sites and one RXR binding site on SIRT1 and their corresponding characteristic binding motif were used for point mutation design. The plasmids of Luc-SIRT1-mut1, Luc-SIRT1-mut2, and Luc-SIRT1-mut3 were amplified by PCR using the Neb Q5 point mutation method. The plasmids of Luc-SIRT1 and VDR overexpression or VDR siRNA were transfected into 293T cells and so were the Luc-Sirt1-mut1, Luc-Sirt1-mut2, and Luc-Sirt1-mut3 plasmids with the VDR overexpression plasmid. After incubation for forty-eight hours, the luciferase activity was evaluated with the dual-luciferase assay system (Promega, #E2920). The primers used in this assay are shown in supplemental Table 2.

### 2.15. Chromatin Immunoprecipitation- (ChIP-) qPCR Assay

HepG2 cells treated with 10^−7^ M 1,25(OH)_2_D_3_ and cultured for 24 hours were collected and cross-linked with 1% formaldehyde (Sigma) for 15 minutes, and ChIP was performed with anti-VDR (Proteintech, #67192-1-Ig) antibody. qRT-PCR was performed as previously described. The primers used in this qPCR assay are shown in supplemental Table 3.

### 2.16. Statistical Analyses

Student's *t*-test was used to compare differences between the analysed samples. A value of *P* < 0.05 was considered statistically significant. Data are expressed as average ± SEM. GraphPad Prism 6.0 was applied to draw graphs.

## 3. Results

### 3.1. The Role of High Calcium and Phosphorus Dietary Supplementation in Correcting Hypocalcemia Caused by 1,25(OH)_2_D_3_ Deficiency

**Figure 1:**
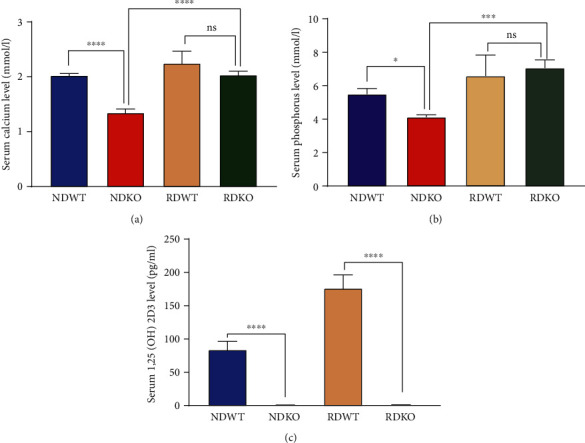
The serum level of calcium, phosphorus and 1,25(OH)_2_D_3_ in each group. Levels of serum calcium (a), phosphorus (b), and 1,25(OH)_2_D_3_ (c) of 1*α*(OH)ase^−/−^ and WT male mice aged 6 months fed with normal diet (ND) or rescue diet (RD). ^∗^*P* < 0.05, ^∗∗∗^*P* < 0.001, ^∗∗∗∗^*P* < 0.0001. ns: not significant; blue bar: NDWT group; red bar: NDKO group; yellow bar: RDWT group; green bar: RDKO group; *n* = 6‐10.

The levels of serum calcium, phosphate, and 1,25(OH)_2_D_3_ in 1*α*(OH)ase^−/−^ mice fed with a ND were significantly lower than those in their WT littermates. To rule out the possible influence of hypocalcaemia on glucose homeostasis, a RD rich in calcium and phosphorus was used to feed WT and 1*α*(OH)ase^−/−^ mice until 6 months of age. No significant changes were found in the serum calcium or phosphorus levels (Figures 1(a) and 1(b)), while serum 1,25(OH)_2_D_3_ was undetectable in the 1*α*(OH)ase^−/−^ mice (Figure 1(c)). These results suggest that a high calcium phosphorus diet can correct hypocalcemia caused by deficiency of active vitamin D, excluding the influence of this factor on the development and progression of glucose metabolism disorder induced by deficiency of active vitamin D.

### 3.2. 1,25(OH)_2_D_3_ Deficiency Increased Hepatic Gluconeogenesis

**Figure 2:**
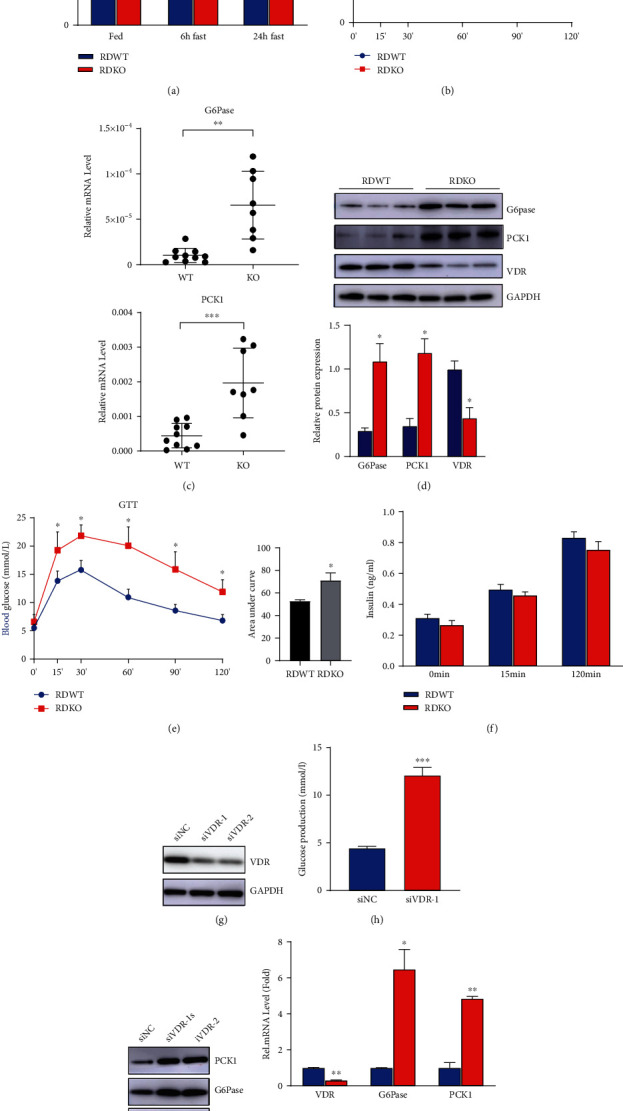
1,25(OH)_2_D_3_ deficiency increases hepatic gluconeogenesis. (a) Blood glucose levels of 6-month-old male 1a(OH)ase^−/−^ and wild-type mice with rescued diet at different time points (fasting, fasting for 6 h, fasting for 24 h) (*n* = 8).^∗^*P* < 0.05; blue bar: RDWT group; red bar: RDKO group. (b) PTT was performed in 6-month-old 1*α*(OH)ase^−/−^ and wild-type mice (*n* = 8). ^∗^*P* < 0.05; blue line: RDWT group; red line: RDKO group. (c) qPCR was used to detect the mRNA expression levels of gluconeogenesis-related genes G6Pase and PCK1 in the liver of 1*α*(OH)ase^−/−^ mice and wild-type littermates (*n* = 8). (d) Western blotting was performed to detect the protein expression levels of G6Pase and PCK1 in the liver of the two groups of mice; blue bar: RDWT group; red bar: RDKO group. (e) GTT was performed in 6-month-old 1*α*(OH)ase^−/−^ and wild-type mice; blue line: RDWT group; red line: RDKO group; *n* = 8. (f) During the GTT, the serum in two groups of mice was collected at 0, 15, and 120 minutes, and the insulin content in the serum of 1*α*(OH)ase^−/−^ mice did not show significant difference as compared to the wild-type littermates; blue bar: RDWT group; red bar: RDKO group; *n* = 8. (g) Western blotting confirmed the siRNA interference efficiency in HepG2 cells. (h) Glucose oxidase method was used to detect the supernatant glucose content of two groups of cells, si-NC group and si-VDR group (*n* = 3). ^∗∗∗^*P* < 0.001. (i, j) Western blotting and qRT-PCR detected the mRNA (j) and protein (i) expressions of G6Pase and PCK1 in the HepG2 cells with VDR knockdown (*n* = 3). ^∗∗^*P* < 0.01; blue bar: RDWT group; red bar: RDKO group; blue line: RDWT group; red line: RDKO group; blue bar: si-NC group; red bar: si-VDR group.

To clarify the role of VD in glucose metabolism, the blood glucose levels were detected and results showed markedly higher blood glucose level in the 1*α*(OH)ase^−/−^ mice than in WT littermates under both feeding and fasting conditions (Figure 2(a)). Subsequently, a PTT was performed to elucidate whether the elevated blood glucose level in 1*α*(OH)ase^−/−^ mice was caused by glucose overproduction in the liver. The results showed that 1*α*(OH)ase^−/−^ mice displayed higher blood glucose levels after pyruvate administration (Figure 2(b)). Consistently, the expression of G6pase and PCK1, two gluconeogenetic genes, showed a 2- to 3-fold increase at both mRNA and protein levels (Figures 2(c) and 2(d)). 1*α*(OH)ase^−/−^ mice also displayed glucose intolerance during the GTT (Figure 2(e)). However, we did not find a significant difference in insulin secretion between 1*α*(OH)ase^−/−^ mice and their WT littermates during the GTT (Figure 2(f)). Consistently, HepG2 cells with VDR knockdown (Figure 2(g)) produced approximately 2-folds more glucose than control cells (Figure 2(h)). The mRNA and protein expression levels of G6Pase and PCK1 also significantly increased in the VDR-knockdown group (Figures 2(i) and 2(j)). Taken together, these results demonstrate that hepatic gluconeogenesis can be enhanced by 1,25(OH)_2_D_3_ deficiency or the knockdown of its receptor.

### 3.3. 1,25(OH)_2_D_3_ Deficiency Increased Gluconeogenesis and Hepatic Insulin Resistance Potentially through Impairing the Phosphorylation of AKT and FOXO1

**Figure 3:**
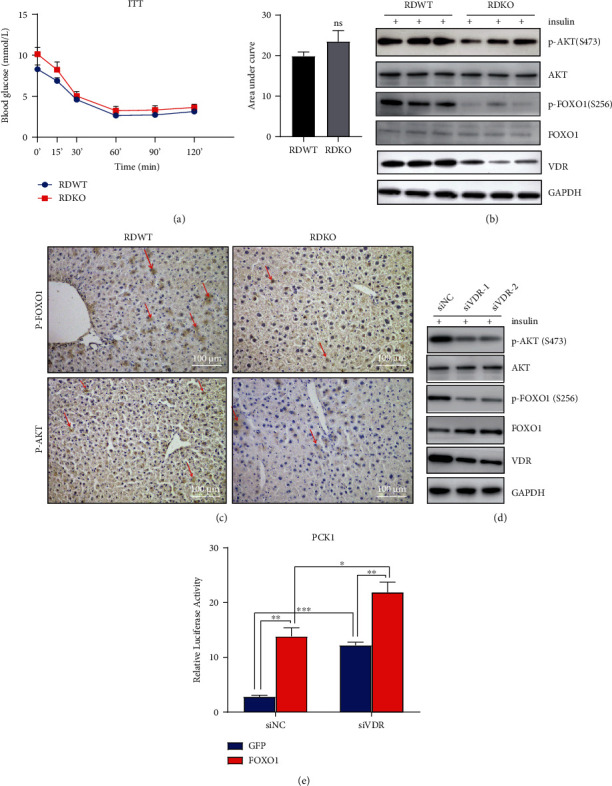
1,25(OH)_2_D_3_ deficiency leads to increased gluconeogenesis and hepatic insulin resistance through impairing phosphorylation of AKT and phosphorylation of FOXO1. (a) ITT was performed in 6-month-old 1*α*(OH)ase^−/−^ and wild-type mice (*n* = 8). ns: not significant; blue line: RDWT group; red line: RDKO group; black bar: RDWT group; gray bar: RDKO group. (b, c) Insulin stimulated the phosphorylation of AKT-S473 and FOXO1-S256 in wild-type livers markedly but failed to do so in the mutant ones, as measured by Western blotting (b) and IHC (c). ^∗^*P* < 0.05. Scale bar = 100 *μ*m; magnification: 20x (c). (d) The phosphorylation of AKT-S473 and FOXO1-S256 was also inhibited in HepG2 cells transfected with si-VDR compared with HepG2 cells transfected with si-NC. ^∗^*P* < 0.05. (e) Ectopic overexpression of FOXO1 increases transcriptional activity of PCK1 promoter. This effect was enhanced by knockdown of VDR. HepG2 cells were transfected with PCK1-luc, together with a FOXO1 expression vector (FOXO1) or a GFP expression vector as a control (GFP). ^∗^*P* < 0.05, ^∗∗^*P* < 0.01, ^∗∗∗^*P* < 0.001.

The liver's response to insulin is essential in suppressing glucose production by lowering down the transcription of PCK1 and G6pase after a meal under normal physiological condition [16, 17]. To clarify whether 1*α*(OH)ase deletion would reduce insulin sensitivity, an ITT was performed, but results showed no difference in the decline in glucose levels upon insulin injection between 1*α*(OH)ase^−/−^ mice and control mice (Figure 3(a)), indicative of normal peripheral insulin responsiveness in 1*α*(OH)ase^−/−^ mice. Insulin has been shown to repress most hepatic genes, which is mostly mediated through a phosphoinositide 3-kinase (PI3K) pathway [18–20]. To further test hepatic insulin sensitivity, the control and mutant animals that had been fasted for 6 hours were treated with insulin, and their livers were collected 30 minutes later. Results showed insulin markedly stimulated the phosphorylation of AKT at S473 in WT mouse livers but failed to do so in the mutant ones, as measured by Western blotting (Figure 3(b)) and immunohistochemistry (IHC) (Figure 3(c)), suggesting hepatic insulin resistance in 1*α*(OH)ase^−/−^ mice. Meanwhile, HepG2 cells transfected with si-VDR also failed to exhibit insulin-mediated activation of AKT/FOXO1 signaling *in vitro* (Figure 3(d)).

Given the above findings, the mechanism by which AKT regulates the expression of G6pase and PCK1 was further investigated. AKT modulates gluconeogenesis via directly phosphorylating FOXO1 at S256, causing its nuclear exclusion [21, 22]. Figures 3(b) and 3(c) showed a dramatically reduced expression of p-FOXO1 S256 in the livers of 1*α*(OH)ase^−/−^ mice as compared to WT mice (Figures 3(b) and 3(c)). Consistently, insulin-induced phosphorylation of FOXO1 at S256 was also reduced in the HepG2 cells with VDR deletion as compared to control HepG2 cells, as revealed by Western blotting (Figure 3(d)).

In the livers of 1*α*(OH)ase-deletion mice, reduced phosphorylation of FOXO1 at S256 suggests increased activity of FOXO1, which has been proven to be able to enhance the transcription of G6pase and Pepck genes [23, 24]. In HepG2 cells, the ectopic expression of FOXO1 activated the PCK1 promoter using a luciferase reporter construct for the PCK1 gene (Figure 3(e)). Consistent with the above findings showing that 1,25(OH)_2_D_3_ deficiency enhances the activity of FOXO1, the ablation of VDR by siRNA, together with the overexpression of FOXO1, synergistically enhanced the above effect (Figure 3(e)). Together with our finding that the transcription of G6pase and PCK1 increased in the 1,25(OH)_2_D_3_-deficient mouse livers, we speculate that the activation of FOXO1 was responsible for the glucose overproduction observed in the livers of 1*α*(OH)ase-knockout mice. Thus, it can be concluded that impaired VD signaling may induce hepatic gluconeogenesis and hepatic insulin resistance through attenuating the activity of the AKT/FOXO1 axis.

### 3.4. Sirt1 Mediated the Effects of 1,25(OH)_2_D_3_ Deficiency on AKT/FOXO1 Signaling and Dysregulation of Glucose Metabolism through Affecting Rictor Transcription

**Figure 4:**
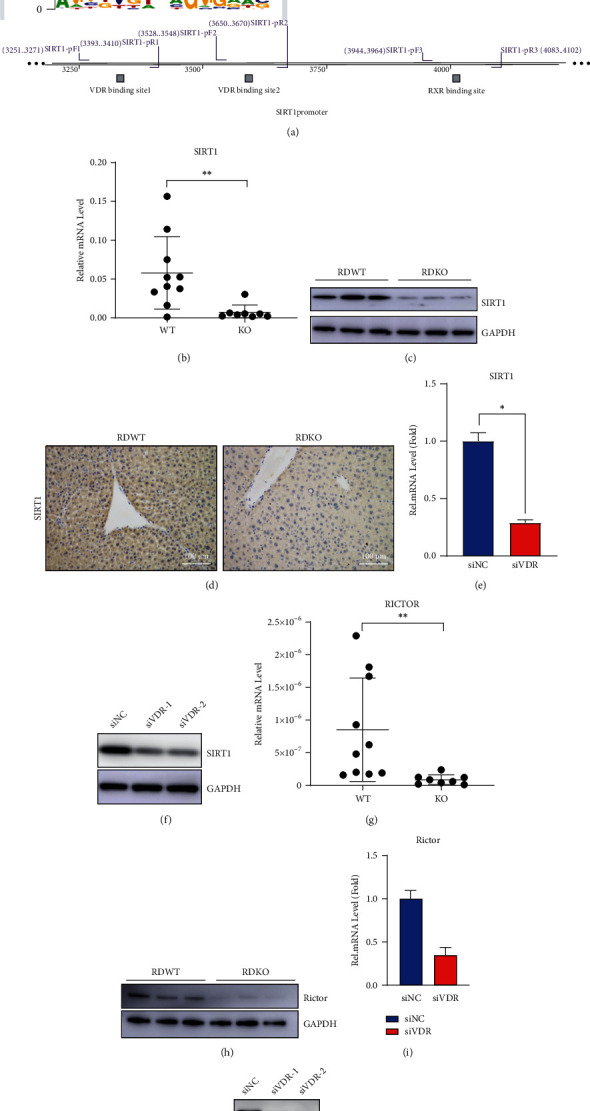
1,25(OH)_2_D_3_ deficiency inhibits the expression of Sirt1, a target gene of VDR/RXR, which then has an inhibitory effect on the Rictor expression. (a) Bioinformatics prediction of binding sites between VDR and Sirt1 (the first and second binding regions in Sirt1 promoter are VDR potential target binding sites and the third binding region in Sirt1 promoter is RXR potential target binding sites). (b, c) mRNA (b) and protein (c) expression levels of Sirt1 in the liver samples collected from wild-type and 1*α*(OH)ase^−/−^ mice with rescued diet. ^∗^*P* < 0.05. (d) IHC was employed to detect the Sirt1 expression in the livers of wild-type and 1*α*(OH)ase^−/−^ mice with rescued diet. Scale bar = 100 *μ*m. (e, f) mRNA (e) and protein (f) expression levels of Sirt1 after transfection of si-NC or si-VDR in HepG2 cells. ^∗^*P* < 0.05; blue bar: si-NC group; red bar: si-VDR group. (g, h) mRNA (g) and protein (h) expression levels of Rictor in the livers from wild-type and 1*α*(OH)ase^−/−^ mice with rescued diet. ^∗^*P* < 0.05, RDWT vs. RDKO. (i, j) mRNA (i) and protein (j) expression levels of Rictor after transfection of si-NC or si-VDR in HepG2 cells. ^∗^*P* < 0.05, si-NC vs. si-VDR; blue bar: si-NC group; red bar: si-VDR group.

**Figure 5:**
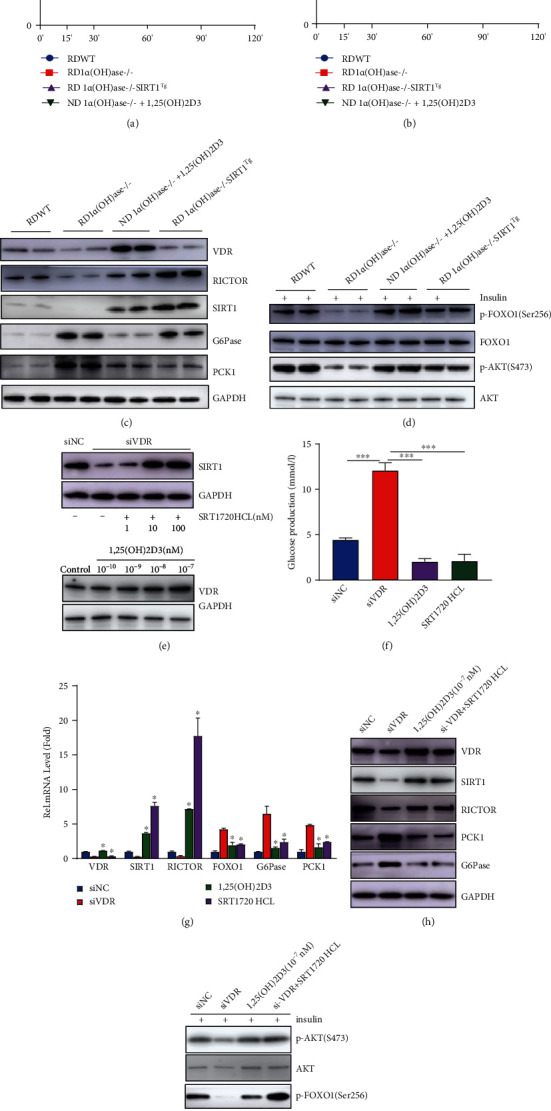
Supplementation of 1,25(OH)_2_D_3_ or overexpression of Sirt1 alleviates hepatic glucose overproduction and glucose metabolism disorders caused by 1,25(OH)_2_D_3_ deficiency. (a) PTT performed in mice of different treatment groups. Blue line: RDWT group; red line: RDKO group; purple line: RD 1*α*(OH)ase-/-SIRT1Tg group; green line: ND 1*α*(OH)ase-/-+1,25(OH)2D3 group. (b) GTT performed in mice of different treatment groups. Blue line: RDWT group; red line: RDKO group; purple line: RD 1*α*(OH)ase-/-SIRT1Tg group; green line: ND 1*α*(OH)ase-/-+1,25(OH)2D3 group. (c) Expression levels of proteins including VDR, Sirt1, Rictor, PCK1, and G6PC collected from liver samples of mice of each group under normal feeding conditions. (d) Phosphorylation of AKT-S473 and FOXO1-S256 in the liver of mice stimulated with insulin. (e) VDR and Sirt1 protein expression levels in HepG2 cells treated with different concentrations of 1,25(OH)_2_D_3_ or Sirt1 agonists (SRT1720HCL) were determined by Western blotting. (f) Glucose production content of HepG2 cells with knockdown of VDR or treated with 1,25(OH)_2_D_3_ or SRT1720 HCL in response to pyruvate stimulation. ^∗∗∗^*P* < 0.001; blue bar: si-NC group; red bar: si-VDR group; purple bar: 1,25(OH)_2_D_3_ group; green bar: SRT1720HCL group. (g) Detection of mRNA expression of related genes in the liver of different treatment groups (qRT-PCR). ^∗^*P* < 0.05. (h) Western blotting was performed to detect the expression levels of related proteins in different treatment groups of HepG2 cells. (i) The phosphorylation of AKT-S473 and FOXO1-S256 in HepG2 cell treated with different concentrations of 1,25(OH)_2_D_3_ and Sirt1 agonist (SRT1720HCL) in response to insulin stimulation was measured by Western blotting.

The potential mechanisms through which 1,25(OH)_2_D_3_ regulates AKT/FOXO1 activity was further explored. Sirt1 is known to positively regulate the AKT/FOXO1 signaling pathway [14]. We identified three VDREs in different regions of the Sirt1 promoter by bioinformatics analysis (Figure 4(a)); thus, we investigated whether VD could change Sirt1 activity at the transcriptional level in the liver. Both the mRNA and protein expression levels of Sirt1 in the livers of 1*α*(OH)ase^−/−^ mice were downregulated as compared to those in WT littermates (Figures 4(b)–4(d)), and similar results were observed in HepG2 cells in which VDR had been silenced (Figures 4(e) and 4(f)). Previous studies have shown that Rictor, an integral component of the mTorc2 complex, can negatively regulate hepatic glucose production and that AKT can be phosphorylated at S473 by the mTorc2 complex in response to stimulation of growth factor [25, 26]. As shown in the study of Wang et al., SIRT1 controls the expression of Rictor through interaction with NRF1 on the NRF1-binding sites of Rictor promoter, thus modulating glucose metabolism and insulin sensitivity through the AKT/FOXO1 axis [14]. Thus, whether Rictor takes part in the regulation of attenuated activity of the AKT/FOXO1 pathway in the livers of 1,25(OH)_2_D_3_-deficient mice was further investigated. Results showed that both the mRNA and protein expressions of Rictor were markedly downregulated in the livers of 1*α*(OH)ase^−/−^ mice as compared to their WT littermates (Figures 4(g) and 4(h)); a consistent trend regarding Rictor expression was also observed in HepG2-si-VDR cells (Figures 4(i) and 4(j)). Meanwhile, the mTOR2 protein expression showed no difference between the above groups, implying Rictor functions as the effective unit. To further test the role of Sirt1 in this axis, we bred 1*α*(OH)ase^−/−^Sirt1^Tg^ mice to specify whether overexpression of Sirt1 could normalize the glucose intolerance observed in 1*α*(OH)ase^−/−^ mice. In comparison to those from 1*α*(OH)ase^−/−^ mice, the liver samples of 1*α*(OH)ase^−/−^Sirt1^Tg^ mice showed increased Sirt1 and Rictor protein expression (Figure 5(c)), and the tolerance of 1*α*(OH)ase^−/−^Sirt1^Tg^ mice to pyruvic acid stimulation (Figure 5(a)) and glucose (Figure 5(b)) was partially improved, as revealed by the PTT and GTT. Meanwhile, 1*α*(OH)ase^−/−^Sirt1^Tg^ mouse livers exhibited enhanced pAKT-S473 and pFOXO1-S256 expression in response to insulin compared to that in the livers of 1*α*(OH)ase^−/−^ mice (Figure 5(d)), suggesting that overexpression of Sirt1 restored the impaired hepatic insulin signaling pathway induced by 1,25(OH)_2_D_3_ deficiency *in vivo*.

In addition, Figure 5(e) shows a marked elevation in Sirt1 expression in HepG2 cells treated with 100 nM SRT1720HCL for 6 hours compared to control cells (Figure 5(e)). Therefore, HepG2 cells were treated with 100 nM SRT1720HCL, and the changes in pyruvate- and lactate-stimulated glucose production and related gene expression were observed. Consistent with the results observed in animals, the administration of SRT1720HCL enhanced the expression of Rictor as well as the FOXO1 target genes G6pase and PCK1, as measured by qRT-PCR (Figure 5(g)) and Western blotting (Figure 5(h)). Meanwhile, increased insulin-stimulated phosphorylation of AKT at S473 and FOXO1 at S256 (Figure 5(i)) and reduced pyruvate- and lactate-stimulated glucose production were also observed in the HepG2 cells treated with SRT1720HCL (Figure 5(f)). Thus, these findings demonstrate that Sirt1 and downstream Rictor are required for VD-mediated induction of FOXO1 nuclear exclusion to control hepatic gluconeogenesis and glucose metabolism.

### 3.5. VDR Positively Regulated the Expression of Sirt1 through Binding to the VDRE in the SIRT1 Promoter

**Figure 6:**
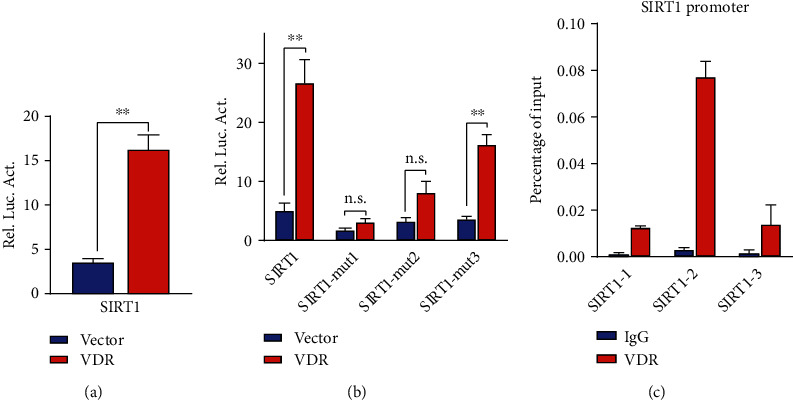
VDR positively regulates the expression of Sirt1 through binding to the VDRE in the SIRT1 promoter. (a) Luciferase reporter assay confirms that overexpression of VDR positively regulates the mRNA expression of Sirt1. ^∗∗^*P* < 0.01. Blue bar: vector group; red bar: VDR group. (b) ChIP-qPCR detection verifies a direct binding between VDR and Sirt1 DNA promoter. Blue bar: vector group; red bar: VDR group. (c) Three specific mutation plasmids were constructed, and the results of luciferase reporting assays show that the third binding region in RXR is not functional, indicating the efficient binding relationship just exists between VDR and Sirt1 promoter. The plasmids of Luc-SIRT1 and VDR overexpression plasmid or VDR siRNA were transfected into 293T cells so were the Luc-Sirt1-mut1, Luc-Sirt1-mut2, and Luc-Sirt1-mut3 plasmids with VDR overexpression plasmid. ^∗∗^*P* < 0.01; ns: not significant; using Student's *t*-test. The experiments were repeated more than 3 times. Sirt1-mut1: transfected with Luc-Sirt1-mut1; Sirt1-mut2: transfected with Luc-Sirt1-mut2; Sirt1-mut1: transfected with Luc-Sirt1-mut3; blue bar: IgG group; red bar: VDR group.

1,25(OH)_2_D_3_, a nuclear hormone, regulates gene transcription and exerts its effects through binding its receptor, VDR [27]. To determine whether VD exerts its effect through binding its receptor to directly modulate Sirt1 expression, a luciferase reporter gene assay was performed and results showed that VDR overexpression significantly enhanced the activity of the Sirt1 promoter while VDR ablation by siRNA conversely inhibited it, indicating a positive regulation between VDR and Sirt1 (Figures 6(a) and 6(b)). As three VDREs were identified in different regions of the Sirt1 promoter by bioinformatics analysis, we next explored which one functions mostly. After site-specific mutation of VDR and RXR binding sites in Sirt1 promoter region, VDR overexpression could still significantly enhance the level of the Sirt1-mut3 promoter but had no significant enhancement effect on the level of Sirt1-mut1 and Sirt1-mut2 promoter (in VDR) (Figure 6(b)), indicating that the mut3 sequence (in RXR) is not a functional region. Meanwhile, a ChIP-qPCR assay in HepG2 cells verified that VDR was physically enriched at the Sirt1 promoter region (Figure 6(c)). Thus, the above findings suggest that VDR positively regulates the expression of Sirt1 at the transcriptional level through binding to the VDRE in the SIRT1 promoter. According to the above findings, either 1,25(OH)_2_D_3_ deficiency or inhibition of VDR leads to the impairment of the Sirt1-mTOR2-AKT/FOXO1 signaling pathway both *in vivo* and *in vitro*. Thus, to the best of our knowledge, these findings for the first time show that Sirt1, a target gene of VDR, directly mediates the influence of impaired VD signaling on the hepatic glucose production and insulin sensitivity.

### 3.6. Supplementation with 1,25(OH)_2_D_3_ Reversed the Phenotype of 1*α*(OH)ase^−/−^ Mice

**Figure 7:**
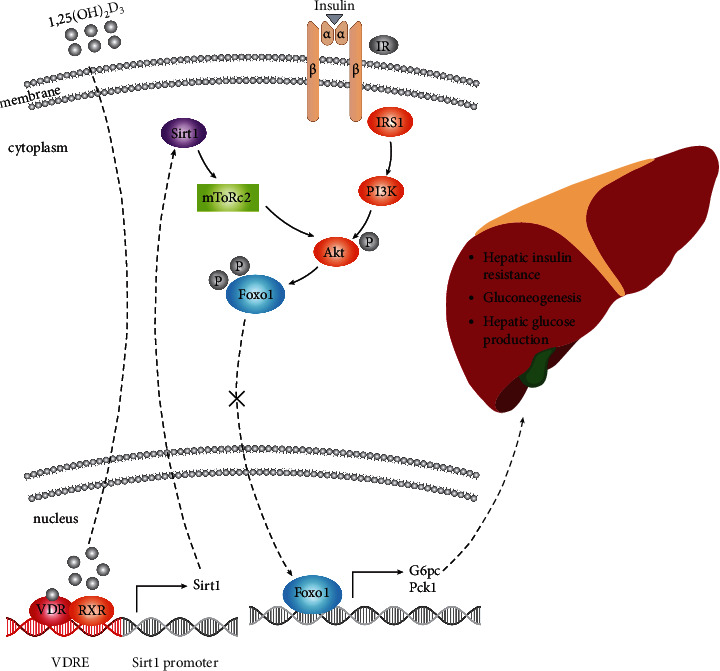
Model of mechanisms leading from 1,25(OH)_2_D_3_ deficiency to increased hepatic gluconeogenesis. 1,25(OH)_2_D_3_-mediated pathway and insulin-mediated pathway converge on AKT, working together to promote AKT phosphorylation at Ser473 and then FOXO1 phosphorylation at Ser256. 1,25(OH)_2_D_3_ deficiency suppresses Sirt1 expression, leading to downregulation of Rictor which further impaired phosphorylation of AKT-S473 and FOXO1-S256. As a result, the expression of G6Pase and PCK1 is upregulated, causing hepatic glucose overproduction and hepatic insulin resistance.

Subsequently, 1,25(OH)_2_D_3_ to 1*α*(OH)ase^−/−^ mice with administered with 1,25(OH)_2_D_3_ to explore whether supplementation with 1,25(OH)_2_D_3_ could reverse the glucose metabolism phenotype of the 1,25(OH)_2_D_3_-deficient mice. The GTT and PTT revealed an improvement in the response to pyruvic acid (Figure 5(a)) or glucose (Figure 5(b)) stimulation. Western blotting revealed that the administration of 1,25(OH)_2_D_3_ enhanced VDR and Sirt1 expression in the livers of 1*α*(OH)ase^−/−^ mice fed with a ND; furthermore, Rictor expression was significantly upregulated, while the expression of key gluconeogenesis genes G6Pase and PCK1 was markedly downregulated as compared to their levels in the 1*α*(OH)ase^−/−^ littermates on a RD (Figure 5(c)). In addition, insulin failed to phosphorylate AKT at S473 and FOXO1 at S256 in the livers of 1*α*(OH)ase^−/−^ mice but could do so in the livers of 1*α*(OH)ase^−/−^ mice supplemented with 1,25(OH)_2_D_3_ (Figure 5(d)), suggesting that 1,25(OH)_2_D_3_ administration improved the impaired hepatic insulin signaling pathway. Consistently, in HepG2 cells treated with 1,25(OH)_2_D_3_, qRT-PCR (Figure 5(g)) and Western blotting (Figure 5(h)) also revealed the reduced expression of FOXO1 target genes G6pase and PCK1. Meanwhile, insulin-stimulated levels of AKT phosphorylated at S473 and FOXO1 phosphorylated at S256 (Figure 5(i)) increased and pyruvate-stimulated glucose production significantly reduced (Figure 5(f)). The above observations demonstrate that enhanced VDR signaling by 1,25(OH)_2_D_3_ promotes FOXO1 phosphorylation and nuclear exclusion in the hepatocytes through positively regulating SIRT1 expression and subsequently activating the mTOR2-AKT/FOXO1 pathway (Figure 7).

## 4. Discussion

Epidemiological investigations have clearly shown that VD deficiency is positively correlated with the occurrence and progression of the metabolic syndrome [28]. However, the specific mechanism by which 1,25(OH)_2_D_3_ deficiency causes hyperglycaemia and insulin resistance remains elusive. Abnormal hepatic glucose production has been implicated as a major contributor to several human diseases, such as liver cirrhosis, insulin resistance, and type 2 diabetes. Furthermore, Sirt1 activity in the liver affects gluconeogenesis and insulin resistance [14]. However, whether VD-deficient signaling in the liver increases gluconeogenesis and insulin resistance or triggers the dysregulation of Sirt1 activity is unknown. Our results showed that 1*α*(OH)ase^−/−^ mice had enhanced gluconeogenesis and hepatic insulin resistance and decreased expression of Sirt1 in the liver. In contrast, enhanced VDR signaling by 1,25(OH)_2_D_3_ upregulated Sirt1 expression in the 1*α*(OH)^−/−^ mice and HepG2 cells. The VD3-dependent activation of Sirt1 expression disappeared when VDR was knocked down. These results suggest that Sirt1 is a major target that mediates 1*α*(OH)ase-null signaling in the liver. Administration of Sirt1 agonist SRT1720 HCL normalized the gluconeogenesis and hepatic insulin sensitivity and prevented the glucose and pyruvate intolerance that occurred in 1*α*(OH)ase^−/−^ mice. These novel findings indicate that suppression of Sirt1 expression in the liver may be responsible for the enhanced gluconeogenesis, hepatic insulin resistance, and impaired glucose metabolism in the 1*α*(OH)ase^−/−^ mice.

Hepatic insulin signaling plays a key role in the gluconeogenesis and glucose metabolism. FOXO1 is a critical target that mediates VDR signaling in the skeletal muscle [10]. Under physiological conditions, insulin inhibits FOXO1 activity by activating the insulin receptor (IR)/phosphatidylinositol-3-kinase (PI3K)/AKT signaling pathway, which induces the phosphorylation of FOXO1 and its transportation to the cytoplasm and degradation, reducing G6Pase and PCK1 expression, diminishing hepatic glucose production, and elevating glycogen synthesis [9, 29, 30]. However, in the case of VD deficiency, the expression of gluconeogenesis-related genes G6pase and PCK1 increased in the 1,25(OH)_2_D_3_-deficient mice and HepG2 cells with VDR knockdown, demonstrating that impaired VD signaling (1*α*(OH)ase deletion or VDR knockdown) may increase hepatic gluconeogenesis and thus result in hyperglycaemia. In addition, our results indicate a significant decrease in the phosphorylation of AKT (S473) and FOXO1 (S256) in the livers of 1*α*(OH)ase^−/−^ mice, increasing FOXO1 accumulation in the nucleus and enhancing the expression of downstream PCK1 and G6Pase. Our results clearly show that 1,25(OH)_2_D_3_ negatively regulates FOXO1 expression and activity in the liver (Figure 3). 1,25(OH)_2_D_3_ activates the AKT/FOXO1 axis, providing evidence of a mechanism linking gluconeogenesis to FOXO1.

Several studies have shown that decreased insulin/PI3K/AKT signaling inhibits FOXO1 phosphorylation at the three sites (T24, S256, and S319), enhancing the subsequent nuclear translocation and activity of FOXO1, inducing insulin resistance [8]. The VD- and insulin-mediated pathways converge on AKT, working together to promote AKT phosphorylation at S473 and subsequent FoxO1 phosphorylation at S256. As shown in Figure 3(b), 1*α*(OH)ase^−/−^ mice had impaired AKT phosphorylation under insulin treatment. However, as shown in Figure 3(a), the ITT failed to show a significant difference in insulin tolerance between WT and KO mice, which indicates that the impairment of insulin-induced AKT phosphorylation in 1*α*(OH)ase^−/−^ mice fails to affect insulin activity. In addition, no abnormalities in serum insulin levels were detectable in the normal and mutant groups, suggesting that the enhanced hepatic gluconeogenesis induced by 1,25(OH)_2_D_3_ deficiency may not cause whole-body insulin resistance in mice aged 6 months; however, we cannot exclude the possibility that skeletal muscle and adipose tissues will also exhibit insulin resistance similar to that in the liver as the 1,25(OH)_2_D_3_-deficient mice grow older. Studies that investigate the biological mechanism by which VD modulates hepatic gluconeogenesis free from insulin are ongoing.

Previous studies have shown that Sirt1, an important gene that regulates glucose and lipid metabolism, can regulate the levels of AKT/FOXO1 phosphorylation through the mediation of Rictor [14, 31]. Given that 1,25(OH)_2_D_3_ deficiency downregulated the expression of Sirt1 and Rictor, we further bred 1*α*(OH)ase^−/−^Sirt1^Tg^ mice and found that the tolerance to glucose and pyruvic acid of these mice was improved as compared to that of WT littermates, suggesting restored glucose metabolism and reduced glucose production. Notably, the activation of the VD signaling pathway by 1,25(OH)_2_D_3_ or the upregulation of Sirt1 through Sirt1^Tg^ or chemical activation by a Sirt1 agonist (SRT1720 HCL) normalized the glucose overproduction and glucose metabolism *in vivo* and *in vitro* through upregulating the AKT phosphorylation at S473 and FOXO1 phosphorylation at S256, leading to reduced expression of PCK1 and G6Pase. However, it remains to be elucidated whether impaired VD/VDR signaling inhibits Sirt1 transcription by direct binding to VDR elements in the Sirt1 promoter, indirect binding to DNA via some contiguous transcription factors, or indirect modulation of other genes.

VDR, a member of the nuclear receptor superfamily, can regulate gene transcription by binding VD-responsive elements in the promoter region of target genes. In most cases, VDR and RXR form a complex that serves to positively regulate gene expression [32]. Therefore, a VDR ChIP assay was performed on the Sirt1 promoter regions, and results showed VDR enrichment in the VDRE of Sirt1 promoter and confirmed the positive regulation between VDR and Sirt1, demonstrating that VDR can directly regulate Sirt1 gene expression at the transcriptional level. Meanwhile, our findings showed that VDR was bound to the VDRE in the Sirt1 promoter and enhanced Sirt1 transcription, which are consistent with the positive role of VDR in gene expression. Study has reported that young liver-specific Sirt1-knockout mice suffer from hyperglycaemia and gradually develop an impaired insulin response as they grow up, finally exhibiting whole-body insulin resistance [33–37]. Consistent with these results, our study indicated that 1,25(OH)_2_D_3_ deletion negatively regulated the mTOR2/AKT pathway by directly inhibiting the transcription of Sirt1 and subsequent Rictor, thereby enhancing hepatic glucose production and causing hyperglycaemia as well as glucose metabolism dysfunction. These novel findings indicate that Sirt1 reduction in the liver may be responsible for the hepatic insulin resistance and impaired glucose metabolism in the 1,25(OH)_2_D_3_-deficient mice. Although our findings suggest that VDR regulates Sirt1 at the transcriptional level, we cannot exclude the possibility that there is posttranscriptional modification of Sirt1. Mutt et al. [38] reported that long-term VD deficiency caused hepatic insulin resistance through impairing the PI3K-AKT-FOXO1 pathway while VD ameliorated the above dysfunction via activating the AKT/GSK3*β* and AKT/FOXO1 signaling pathways, which was consistent with our findings. However, they did not further investigate the underlying mechanisms between VD deficiency and impaired activation of the AKT-FOXO1 pathway. In our study, we, for the first time, identified VDR-Sirt1 as the key genes mediating the pathology and the downstream critical canonical AKT/FOXO1 pathway.

As reviewed by Bouillon et al., there are several reports that examined the effects of vitamin D signaling on glucose metabolism [39]. Some reports show positive findings, and others are negative. Our study revealed that the serum insulin level of mice with low calcium and low phosphorus caused by vitamin D deficiency did not decrease significantly compared with wild-type mice when fed with high calcium and phosphorus diet, which may be related to the removal of the low calcium due to lack of VD intake. In addition, VDR-null mice are often used for the establishment of VD deficiency models in previous studies. Here, we generated homozygous mice with targeted deletion of the 1*α*(OH)ase gene which still had VDR expression in cells in spite of decreased VDR level, which is in line with the real physiology of VD deficiency in humans. The main pathological manifestations of 1*α*(OH)ase-null mice were glucose overproduction and insulin resistance in the liver in the present study, although the serum insulin did not appear abnormal in the 1,25(OH)_2_D_3_-deficient mice.

## 5. Conclusions

In summary, our study shows that VDR and RXR form a protein complex that binds the VDRE on the Sirt1 promoter, positively regulating Sirt1 expression. 1*α*(OH)ase deletion decreases the expression of Sirt1 and Rictor, causing a reduction in the phosphorylation of AKT at S473. This event maintains the nuclear localization of FOXO1 and induces increases in the transcription and translation of G6pase and PCK1, two gluconeogenetic genes, giving rise to glucose overproduction in the liver and hyperglycaemia and uncovering a mechanism by which VD deficiency eventually leads to chronic hyperglycaemia and hepatic insulin resistance. By understanding how 1,25(OH)_2_D_3_ deletion stimulates G6PC and PCK1 transcription through suppression of the Sirt1-mTOR2/AKT/FOXO1 axis and determining the role of Sirt1 in VD deficiency-induced impairment of insulin sensitivity and glucose intolerance, we provide critical evidence about a significant metabolic pathway involved in the type 2 diabetes.

## Data Availability

The data used to support the findings of this study are available from the corresponding author upon request.

## Supplementary Materials

Supplementary Materials Supplemental Table 1: qRT-PCR primers. Supplemental Table 2: primers used in Luciferase assay. Supplemental Table 3: ChIP-qPCR primers.
