# Supplementary material for: Sirt1 Mediates Vitamin D Deficiency-Driven Gluconeogenesis in the Liver via mTorc2/Akt Signaling
Source: J Diabetes Res. 2022 Jan 29;2022:1755563. doi: 10.1155/2022/1755563 (PMC8817869; doi:10.1155/2022/1755563)
Supplement: Supplementary Materials — Supplemental Table 1: qRT-PCR primers. Supplemental Table 2: primers used in Luciferase assay. Supplemental Table 3: ChIP-qPCR primers. [file 1755563.f1.docx]

# SUPPLEMENTAL INFORMATION

Suppl. Table 1: qRT-PCR primers

| The Origin of Species | Genes | Primers | Sequences (5′–3′) |
| --- | --- | --- | --- |
| Mouse | vdr | Forward | CTTCAACGCTATGACCTGTG |
|  |  | Reverse | CCTCTTCCTCCTTCCTCTTC |
|  | sirt1 | Forward | AAGTTGACCTCCTCATTGTT |
|  |  | Reverse | AAGTTTGGCATATTCACCAC |
|  | Pck1 | Forward | GTCATCATCACCCAAGAGCA |
|  |  | Reverse | TAGGGCGAGTCTGTCAGTTC |
|  | G6pase | Forward | CTCGCTATCTCCAAGTGAAT |
|  |  | Reverse | TGCTGTAGTAGTCGGTGTCC |
|  | Foxo1 | Forward | GATAAGGGCGACAGCAACAG |
|  |  | Reverse | GATTGAGCATCCACCAAGAA |
|  | Rictor | Forward | GAAGCCAGCCTTATCCCACC |
|  |  | Reverse | CTTTGATTACTGCGACGAAC |
|  | Gapdh | Forward | ATGTTTGTGATGGGTGTGAA |
|  |  | Reverse | ATGCCAAAGTTGTCATGGAT |
| Human | SIRT1 | Forward | CAAAGGAGCAGATTAGTAGG |
|  |  | Reverse | CTGCCACAAGAACTAGAGGA |
|  | VDR | Forward | CCGCATCACCAAGGACAACC |
|  |  | Reverse | TCATCTCCCGCTTCCTCTGC |
|  | PCK1 | Forward | CAGGCAGTGAGGGAGTTTCT |
|  |  | Reverse | TTGCTCTTGGGTGACGATAA |
|  | G6Pase | Forward | CCTACAGATTTCGGTGCTTG |
|  |  | Reverse | GATGCTGTGGATGTGGCTGA |
|  | FOXO1 | Forward | GGATAAGGGTGACAGCAACA |
|  |  | Reverse | GATTGAGCATCCACCAAGAA |
|  | RICTOR | Forward | CCTCATTCACATAGCCATCA |
|  |  | Reverse | GCTTAGGTCCTCGTTTCTTC |

Suppl. Table 2: Primers used in Luciferase assay

| Primers | Sequences (5'-3') |
| --- | --- |
| VDR-EcoRI-F | CCTGAATTCATGGAGGCAATGGCGGC |
| VDR-BamHI-R | CTGGGATCCTCAGGAGATCTCATTGCC |
|  | |
| Luc-nestF | AGTTCCAGGATACAGGTGCAG |
| Luc-nestR | GAAGGAAATGACTTGGCCTCCA |
|  | |
| Luc-SIRT1-NheI-F | TTTGCTAGCAGACGGAGTTTCGCTCTTGCT |
| Luc-SIRT1-HindIII-R | TTAAAGCTTCTCATGTCTTAAATGGTAAT |
| Point mutation PCR primers | |
| SIRT1-mut1-F | TCAACCATCCTGCCTCAGCCTTCTG |
| SIRT1-mut1-R | CGTGGTGAAGCAGGCAGAGGTTGCGG |
|  | |
| SIRT1-mut2-F | CTCAGGATAAAGATGGTTTTAAGGCTTCTAG |
| SIRT1-mut2-R | TATCCAATAGGGGAGGGAGAGCATTA |
|  | |
| SIRT1-mut3-F | CCACCACACTAGGTACCCCTCGTTTTA |
| SIRT1-mut3-R | TCATGGAGGTGGAAAGCCCTTCCACT |

Suppl. Table 3: ChIP-qPCR primers

| Primers | Sequences (5'-3') |
| --- | --- |
| SIRT1-pF1 | AGACGGAGTTTCGCTCTTGCT |
| SIRT1-pR1 | AAAATACAAAATTAGCCG |
| SIRT1-pF2 | AGCCAGGTTTTAAGTCCCGCA |
| SIRT1-pR2 | TTCATCTGGTCACCACTATTC |
| SIRT1-pF3 | AGATATGGAGTCACAGTGTGC |
| SIRT1-pR3 | CTCATGTCTTAAATGGTAAT |
